# Haematological, Biochemical, and Inflammatory Biomarkers of COVID-19 Patients Hospitalized in Critical Unit: A Retrospective Study

**DOI:** 10.7759/cureus.23691

**Published:** 2022-03-31

**Authors:** Radi T Alsafi, Faisal Minshawi, Ahmad Alshareef, Essa Althobiany, Afnan Alqurashi, Ayat Zawawi, Ahmed Qasem, Amr J Halawani, Mohammed Almatrafi, Hassan Alwafi, Mohammed Samannodi, Emad Salawati, Hamza M Assaggaf

**Affiliations:** 1 Laboratory Medicine Department, Faculty of Applied Medical Sciences, Umm Al-Qura University, Mecca, SAU; 2 Clinical Molecular Department, Regional Laboratory in Makkah, Ministry of Health, Mecca, SAU; 3 Laboratory and Blood Bank, Al Noor Specialist Hospital, Ministry of Health, Mecca, SAU; 4 Molecular Biology, Independent Researcher, Mecca, SAU; 5 Department of Medical Laboratory Technology, Faculty of Applied Medical Sciences, King Abdulaziz University, Jeddah, SAU; 6 Department of Pediatrics, Umm Al-Qura University, Mecca, SAU; 7 Pharmacology and Therapeutics, Umm Al-Qura University, Mecca, SAU; 8 Department of Medicine, Faculty of Medicine, Umm Al-Qura University, Mecca, SAU; 9 Family Medicine, King Abdulaziz University, Jeddah, SAU

**Keywords:** sars-cov-2, chronic comorbidities, inflammatory biomarkers, icu, covid-19

## Abstract

Background: The World Health Organization declared coronavirus disease 2019 (COVID-19) responsible for a catastrophic global pandemic. The complexity of COVID-19 is centred on the unpredictable course of the disease, which can rapidly develop from patients being asymptomatic to having life-threatening symptoms. The unpredictable disease severity of the severe acute respiratory syndrome coronavirus 2 (SARS-CoV-2) infection has been a major problem facing the healthcare system during the pandemic. Identifying the laboratory biomarkers would help predict SARS-CoV-2 pathogenicity. This study focused on the previous literature regarding three laboratory biomarker profiles: haematological, inflammatory, and biochemical biomarkers.

Methods: A retrospective study of COVID-19 patients was conducted between May 2020 and September 2020 to determine the predictors of hospitalization (severity) in COVID-19 patients. Patients were divided into two groups: those admitted to an intensive care unit (ICU, severe) and those admitted to a non-ICU (stable). Patients' data were obtained from their medical records at Al Noor Specialist Hospital and East Arafat Hospital in Saudi Arabia.

Results: A total of 487 patients with COVID-19, including 304 males and 183 females, were investigated in this study. A total of 217 patients were admitted to the ICU. Patients admitted to the ICU had a higher prevalence of chronic comorbidities than non-ICU patients. D-dimer, white blood cells (WBC), neutrophils, ferritin, C-reactive protein (CRP), lactate dehydrogenase (LDH), alanine aminotransferase (ALT), and aspartate aminotransferase (AST) were more elevated in patients admitted to the ICU compared to non-ICU patients.

Conclusion: Chronic comorbidities are a significant predictor for admission to the ICU. Moreover, tests for D-dimer, WBC, neutrophils, lymphocytes, CRP, LDH, and ALT could be used to predict patients' admission to the ICU.

## Introduction

The current coronavirus disease 2019 (COVID-19) was first reported in Wuhan, the capital of China’s Hubei province, in December 2019. In February 2020, the World Health Organization (WHO) declared COVID-19 responsible for a catastrophic global pandemic [1,2]. The novel virus among human coronaviruses is called severe acute respiratory syndrome coronavirus 2 (SARS-CoV-2), an enveloped RNA [1,2]. The symptoms of COVID-19 range from asymptomatic to life-threatening symptoms. COVID-19 typically causes headaches, malaise, fever, dry cough, and fatigue, which can quickly progress to shortness of breath, acute respiratory distress syndrome, cardiac injury, septic shock, multi-organ failure, and death [3,4].

Recent studies highlighted the importance of identifying reliable laboratory biomarkers related to COVID-19 progression. For instance, Leulseged et al. found that changes in the white blood cells (WBC) and sodium (Na) levels were significantly associated with severe cases of COVID-19 in developing countries [5]. Perhaps, identifying reliable biomarkers would help to determine the severity of SARS-CoV-2 infection, as well as cell and organ failure. Accurate and reliable biomarkers would also benefit the clinical management of COVID-19. It would enable patients to be categorized into risk groups following diagnosis to guarantee prompt treatment, prevent serious complications, and ensure optimal resource allocation [6,7].

This study primarily investigates the different biomarkers implicated in COVID-19 for their potential application as markers of disease severity. The current study reviewed three categories of laboratory biomarkers, summarized as haematological, biochemical, and inflammatory biomarkers. The haematological biomarkers were WBC, lymphocytes, neutrophils, and platelet count. The inflammatory biomarkers were C-reactive protein (CRP), neutrophil-lymphocyte ratio (NLR), and lactate dehydrogenase (LDH). The biochemical biomarkers included D-dimer, alanine aminotransferase (ALT), and aspartate aminotransferase (AST).

## Materials and methods

Study design

A retrospective study of 487 COVID-19 patients was conducted to determine the predictors of hospitalization (severity) of COVID-19. The patients were divided into two groups: those admitted to an intensive care unit (ICU) and those admitted to a non-ICU. Patients' data were obtained from their medical records at holy Makkah's Al Noor Specialist Hospital and East Arafat Hospital between May 2020 and September 2020. The study focused on the patients' age, nationality, gender, and comorbidities including hypertension, diabetes mellitus, and pulmonary diseases. Patients' status was tracked from the time they were admitted to the hospital until they were discharged.

Statistical analysis

The study used a cross-sectional design and was conducted retrospectively. After analysing the data with Statistical Package for the Social Sciences (SPSS) version 25 (IBM Corp., Armonk, NY), univariate logistic regression and Kruskal-Wallis test were used to determine the association between the variables, as the data were not normally distributed. The study included multiple populations and independent samples, and the data were non-parametric. Following data analysis, the results were presented in numbers, percentages, and p-values (p < 0.05), which were used to interpret the results.

Ethical approval

Ethical approval was obtained from the Internal Review Board of the Security Forces Hospital Makkah (SFHM) (# 0432-280621).

## Results

Characteristics of the study population

The clinical assessment of 487 patients with COVID-19 was integrated into the present study (Table 1), including 304 males and 183 females. The median age was 55 ± 14 years. A total of 217 patients were admitted to the ICU. The mean age of the patients admitted to the ICU was 57 ± 13 years, which is higher than that of non-ICU patients with a mean age of 53 ± 15 years (p-value: 0.001). In addition, results showed the number of patients with chronic comorbidities, such as hypertension and diabetes mellitus, in ICU was 13.9% and 20%, respectively. Lastly, according to the hospital's reports, the cause of death of the patients was COVID-19 and the mortality rate among patients admitted to the ICU was 12% due to SARS-CoV-2 infection, which is higher than the non-ICU patients at 1%.

**Table 1:** Demographic and clinical characteristics of ICU and non-ICU COVID-19 patients. * P ≤ 0.05.

	Total (%)	ICU (%)	Non-ICU (%)	P-value
N (%)	487	217 (44.6)	270 (55.4)	
Age, mean ± SD	55 ± 14	57 ± 13	53 ± 15	0.001*
Gender				0.63
Male	304 (62.4)	138 (28.3)	166 (34.1)	
Female	183 (37.6)	79 (16.2)	104 (21.4)	
Nationality				0.37
Saudi	229 (47)	107 (22)	122 (25)	
Non-Saudi	258 (53)	110 (22.6)	148 (30.4)	
Discharge status				0.001*
Dead	68 (14)	63 (13)	5 (1)	
Alive	419 (86)	154 (31.6)	265 (54.4)	
Hypertension				0.001*
Yes	120 (24.6)	68 (13.9)	52 (10.7)	
No	367 (75.4)	149 (30.6)	218 (44.8)	
Diabetes mellitus				0.001*
Yes	185 (38)	102 (21)	83 (17)	
No	302 (62)	115 (23.6)	187 (38.4)	
Pulmonary diseases				0.05*
Yes	243 (49.9)	119 (24.4)	124 (25.2)	
No	244 (50.1)	98 (20.1)	146 (30)	

Evaluation of laboratory tests in patients with SARS‐CoV‐2 infection

All laboratory tests included in this study are shown in Table 2. D-dimer, WBC, neutrophils, ferritin, CRP, NLR, LDH, ALT, and AST were more elevated in patients admitted to the ICU than non-ICU patients. Moreover, our study revealed a statistically significant difference in D-dimer, neutrophils, and lymphocyte count between ICU and non-ICU patients. However, our data showed no significant difference in NLR between the comparison groups.

**Table 2:** Laboratory-based biomarkers of ICU and non-ICU COVID-19 patients (n = 487). Reference range was obtained from the laboratory records. * P ≤ 0.01. ICU: intensive care unit; N = number of patients with available data in hospitals' records; SD: standard deviation; WBC: white blood cell; CRP: C-reactive protein; LDH: lactate dehydrogenase; ALT: alanine aminotransferase; AST: aspartate aminotransferase.

	ICU (n = 217)		Non-ICU (n = 270)		
Analyte (reference range, unit)	N (%)	Mean ± SD	N (%)	Mean ± SD	P-value
D-dimer (<0.5 mg/L)	178 (82)	5.68 ± 14.5	205 (75.9)	1.55 ± 2.6	0.001*
WBC (4.5 to 11.0 × 10^9^/L)	207 (95.4)	10.16 ± 4.8	249 (92.2)	8.59 ± 15.4	0.15
Neutrophils (2.0-8.0 x 10^9^/L)	194 (89.4)	8.56 ± 4.9	191 (70.7)	5.25 ± 3.2	0.001*
Lymphocytes (1.0-4.0 x10^9^/L)	194 (89.4)	1.05 ± 0.8	191 (70.7)	1.38 ± 0.9	0.001*
Platelets (150-400 10^9^/L)	207 (95.4)	248.9 ± 89.4	248 (91.9)	264.9 ± 107.3	0.09
NLR (0.8 and 3.5)	80 (36.9)	18.82 ± 39.6	71 (26.3)	10.2 ± 42.7	0.20
Ferritin (30-300 mg/L)	16 (7.4)	921.3 ± 774.4	30 (11.1)	580.1 ± 485.7	0.07
CRP (<10 mg/L)	39 (18)	12.58 ± 6.7	63 (23.3)	9.17 ± 6.1	0.01*
LDH (140-280 U/L)	132 (60.8)	457.9 ± 202.7	164 (60.7)	335.7 ± 149.1	0.001*
ALT (8-48U/L)	198 (91.2)	56.8 ± 46.1	218 (80.7)	46.7 ± 41.1	0.02*
AST (8-48U/L)	198 (91.2)	66.3 ± 63.1	221 (81.9)	49.7 ± 79.3	0.02*

Regarding the inflammatory markers, ICU patients had significantly higher CRP and LDH. ALT and AST outcomes in ICU patients were 56.8 ± 46.1 U/L and 66.3 ± 63.1 U/L, respectively. Overall, logistic regression analysis showed that D-dimer, WBC, neutrophils, lymphocytes, CRP, LDH, ALT, and AST might be components that could predict ICU admission, as demonstrated in Table 3.

**Table 3:** Logistic regression analysis of COVID-19 among patients infected with SARS-CoV-2 (n = 487). * P ≤ 0.01. SARS-CoV-2: severe acute respiratory syndrome coronavirus 2; WBC: white blood cell; NLR: neutrophil-to-lymphocyte ratio; CRP: C-reactive protein; LDH: lactate dehydrogenase; ALT: alanine aminotransferase; AST: aspartate aminotransferase.

	OR (95% CI)	P-value
D-dimer	0.98 (0.84-0.95)	0.001*
WBC	0.99 (0.96-1.01)	NS
Neutrophils	0.78 (0.73-0.84)	0.001*
Lymphocytes	1.77 (1.30-2.43)	0.001*
Platelets	1.00 (1.00-1.01)	NS
NLR	0.99 (0.98-1.01)	0.27 NS
Ferritin	0.99 (0.98-1.00)	0.10 NS
CRP	0.92 (0.86-0.98)	0.01*
LDH	0.99 (0.98-1.01)	0.00*
ALT	0.99 (0.97-0.99)	0.02*
AST	0.99 (0.97-1.00)	0.03*

## Discussion

The COVID-19 pandemic has caused a significant burden to scientific and medical facilities since 2020. Symptoms of COVID-19 can range from mild and self-limiting to severe and fatal. Predicting who will develop a severe inflammatory reaction requiring ICU admission has been challenging. Therefore, clinicians can use laboratory biomarkers to assist decision-making, interpret clinical symptoms more confidently, provide data of underlying biological processes, start treatment, and assess the disease's progression [7].

This study has analysed a range of COVID-19 biomarkers for their usefulness in indicating COVID-19 severity. We have demonstrated that, compared to other haematological biomarkers, there was a significant increase in neutrophils count and a decrease in lymphocytes count in ICU-admitted COVID-19 patients compared to non-ICU patients. Neutrophil and lymphocyte counts are considered the most clinically relevant biomarkers of the WBC count. However, the role of lymphocytes in upper respiratory tract viral infections and their possible importance in therapeutic strategies are not entirely clear [8]. Multiple studies have reported significant lymphopenia with a consistently high NLR due to high neutrophil count and low lymphocyte count in critically ill COVID-19 patients compared to non-ICU patients [9,10]. However, lymphocyte count may not be an independent biomarker of COVID-19 severity due to various factors, such as glucocorticoid treatment and sepsis from concomitant infections [11]. Therefore, further data collection and research on NLR accuracy as a potential independent biomarker of COVID-19 are necessary.

In the present study, we observed elevation of D-dimer in hospitalized COVID-19 patients, particularly those with severe illness (ICU-admitted patients). A similar finding regarding the elevation of D-dimer was reported by other recent studies [12-14]. Increased D-dimer level in COVID-19 patients admitted to the ICU is comparable to that seen in patients with community-acquired pneumonia [15]. In addition, several studies have also associated D-dimer with an increased risk of respiratory degradation, thrombosis, and death from COVID-19 infection [12-14].

In this report, CRP is high in ICU patients. It has been suggested that CRP is strongly correlated with proinflammatory cytokine levels and interleukin-6 (IL-6) [16]. IL-6 has been known to drive CRP production primarily in the liver and as a driver of the COVID-19 cytokine storm associated with poor COVID-19 outcomes [16-21]. Similarly, it has been reported that fatal outcomes from COVID-19 are accompanied by significant liver injury and elevated LDH, ALT, and AST liver enzymes. Therefore, it has been suggested that liver enzymes might be used to indicate COVID-19 severity [22-24]. A previous meta-analysis showed that elevated CRP, D-dimer, and LDH could predict the high severity of COVID-19 [25].

Limitations of this study include selection bias as the study was conducted at two hospitals in Makkah, Saudi Arabia. The sampling was only from Al Noor Specialist Hospital, the largest hospital in Makkah, and East Arafat Hospital was designated for COVID-19 patients throughout the pandemic. Moreover, this study did not control for other therapeutic medications such as steroids and biological treatments including anti-IL-6 blockers. However, finding changes in biomarker levels in COVID-19 patients may be worthwhile in clinical practice to guide treatment and admission to the ICU. Further research is needed to investigate the predictive value of biomarkers to improve the reliability and reproducibility of COVID-19 studies.

## Conclusions

In summary, this study showed that certain markers such as D-dimer, neutrophils, lymphocytes, CRP, LDH, ALT, and AST could predict ICU admission of the COVID-19 patients. Clinical assessment of COVID-19 patients and thorough identification of potential biomarkers can help the development of therapeutic interventions and improve prognoses. Such stratification will enhance patient and resource management and potentially reduce mortality rates. COVID-19 is a global pandemic; thus, we urge extensive international interventional trials so that the biomarker changes noted in this study and other studies can be better understood.
